# Mild Clinical Presentation of Joubert Syndrome in a Male Adult Carrying Biallelic *MKS1* Truncating Variants

**DOI:** 10.3390/diagnostics11071218

**Published:** 2021-07-06

**Authors:** Raffaella Brunetti-Pierri, Marianthi Karali, Francesco Testa, Gerarda Cappuccio, Maria Elena Onore, Francesca Romano, Giuseppe De Rosa, Enrico Tedeschi, Nicola Brunetti-Pierri, Sandro Banfi, Francesca Simonelli

**Affiliations:** 1 Eye Clinic, Multidisciplinary Department of Medical, Surgical and Dental Sciences, Università degli Studi della Campania ‘Luigi Vanvitelli’, Via Pansini 5, 80131 Naples, Italy; raffaella.brunettipierri@unicampania.it (R.B.-P.); karali@tigem.it (M.K.); giuseppe.derosa@unicampania.it (G.D.R.); francesca.simonelli@unicampania.it (F.S.); 2 Telethon Institute of Genetics and Medicine, Via Campi Flegrei 34, 80078 Pozzuoli, Italy; brunetti@tigem.it (N.B.-P.); banfi@tigem.it (S.B.); 3 Department of Translational Medicine, Federico II University, Via Pansini 5, 80131 Naples, Italy; g.cappuccio@tigem.it; 4 Medical Genetics, Department of Precision Medicine, Università degli Studi della Campania ‘Luigi Vanvitelli’, Via Luigi De Crecchio 7, 80138 Naples, Italy; maria.elena.onore@gmail.com (M.E.O.); romano.fra89@hotmail.it (F.R.); 5 Department of Advanced Biomedical Sciences, Federico II University, Via Pansini 5, 80131 Naples, Italy; enrico.tedeschi@unina.it

**Keywords:** *MKS1*, autosomal recessive Retinitis Pigmentosa, Joubert syndrome, phenotype extension

## Abstract

Pathogenic variants in the *MKS1* gene are responsible for a ciliopathy with a wide spectrum of clinical manifestations ranging from Meckel and Joubert syndrome (JBTS) to Bardet-Biedl syndrome, and involving the central nervous system, liver, kidney, skeleton, and retina. We report a 39-year-old male individual presenting with isolated Retinitis Pigmentosa (RP), as assessed by full ophthalmological evaluation including Best-Corrected Visual Acuity measurements, fundus examination, Goldmann Visual Field test, and full-field Electroretinography. A clinical exome identified biallelic nonsense variants in *MKS1* that prompted post-genotyping investigations for systemic abnormalities of ciliopathy. Brain magnetic resonance imaging revealed malformations of the posterior cranial fossa with the ‘molar tooth sign’ and cerebellar folia dysplasia, which are both distinctive features of JBTS. No other organ or skeletal abnormalities were detected. This case illustrates the power of clinical exome for the identification of the mildest forms of a disease spectrum, such as a mild JBTS with RP in the presented case of an individual carrying biallelic truncating variants in *MKS1*.

## 1. Introduction

Pathogenic variants in *MKS1* are responsible for a group of autosomal recessive ciliopathies, including Meckel-Gruber syndrome 1 (MKS1; OMIM # 249000), Joubert syndrome 28 (JBTS; OMIM # 617121) and the Bardet-Biedl syndrome 13 (BBS; OMIM # 615990) [1]. These conditions can have variable severity, multi-organ involvement, and extensive clinical overlap [2]. MKS is the severest disorder that is often lethal in the prenatal or perinatal period and presents with brain malformations of the posterior fossa such as occipital encephalocele, hepatic ductal plate malformation, cystic dysplastic kidneys, and polydactyly [3]. JBTS is less severe than MKS and is typically characterized by hypotonia in infancy followed by ataxia later in life, intellectual disability, and cerebellar vermis hypoplasia which results in the distinctive ‘molar tooth sign’ on brain Magnetic Resonance Imaging (MRI) [4,5]. Additional clinical features of JTBS include tachypnea and/or apnea, occipital encephalocele, oculomotor apraxia, strabismus, nystagmus, ptosis, chorioretinal coloboma, retinal dystrophy, optic nerve atrophy, hepatic fibrosis, cystic kidney disease, oral hamartomas, endocrine disorders, polydactyly, and small ribcage [4,5,6]. BBS is at the mildest end of the spectrum and presents with cognitive deficit, rod-cone dystrophy, obesity, renal cysts, hypogonadism, and postaxial polydactyly [7].

Although a clear genotype-phenotype correlation has not yet been established, loss-of-function *MKS1* variants have been found to be responsible for approximately 7–13% of MKS cases [8,9], whereas hypomorphic *MKS1* variants have been associated with milder clinical presentations, such as BBS [10] and JBTS [11,12,13,14,15]. As an exception to this correlation, biallelic truncating *MKS1* variants have been detected in two JBTS individuals [16,17]. We report a further milder phenotype in an individual with adult-onset Retinitis Pigmentosa (RP) who was found to harbor biallelic nonsense variants in the *MKS1* gene. Reverse phenotyping revealed the presence of the ‘molar tooth sign’ but no other systemic defects.

## 2. Case Presentation

A 39-year-old Caucasian male subject was referred to the Eye Clinic, Multidisciplinary Department of Medical, Surgical and Dental Sciences, Università degli Studi della Campania ‘Luigi Vanvitelli’ for night blindness and decreased visual acuity. He was the first of two siblings born to non-consanguineous healthy parents and his younger sister did not have vision complains. He was born by caesarean section after 38 weeks of an uncomplicated pregnancy with a birth weight of 3000 g (11th pc). He walked independently and he pronounced his first words at about 2 years of age. During infancy he had some speech difficulties that required speech therapy. He attended school with a mild learning difficulty, but he graduated from high school, he lives independently and is employed as an informatic technician. At the age of 39 years, his weight was 81.9 kg (96th centile), his height was 172.5 cm (45th centile), and his occipito-frontal circumference was 56.5 cm (48th centile).

**Figure 1:**
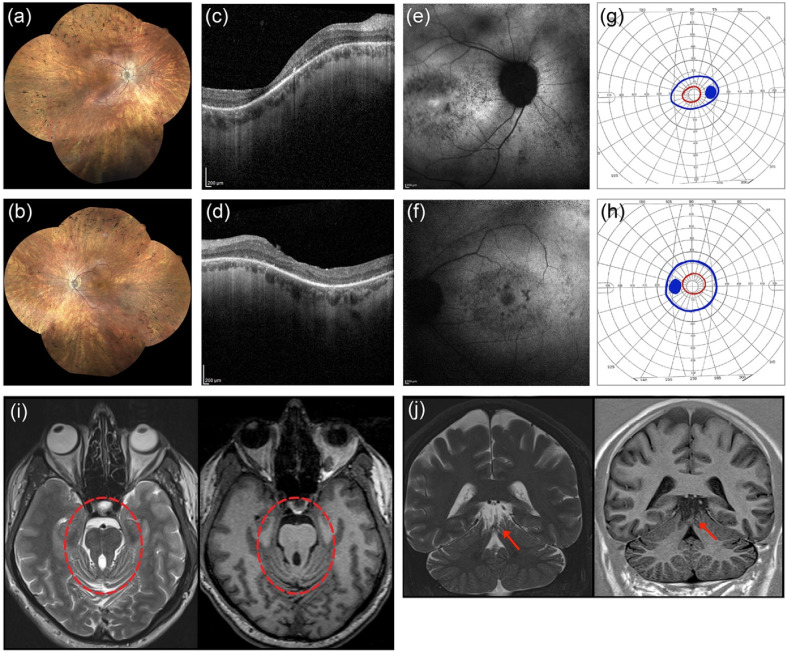
Clinical findings in the proband. Ophthalmological findings in the right eye (RE) are shown in panel (**a**,**c**,**e**,**g**), and in the left eye (LE) in (**b**,**d**,**f**,**h**). Fundus imaging of the proband (**a**,**b**) revealed a waxy pallor of the optic disc with a circumpapillary atrophy, and widespread dystrophy of the retinal pigment epithelium with bone spicule-shaped pigment deposits in mid-periphery. Optical coherence tomography (**c**,**d**) showed reduced macular thickness and RPE dystrophy in both eyes and a vitreoretinal interface syndrome in the LE. Fundus autofluorescence imaging (**e**,**f**) showed focal areas of hypoautofluorescence at the posterior pole with foveal hyperautofluorescence in the RE and foveal hypoautofluorescence in the LE. Visual field tests (**g**,**h**) showed a bilateral constriction of the III4e (blue color) and V4e isopters field (red color). Axial (**i**) and coronal (**j**) T2- (left side) and T1-weighted (right side) brain MRI images at the level of the upper vermis and mesencephalon. The abnormally thickened and elongated superior cerebellar peduncles resemble the shape of a molar tooth on the axial images (red circle). The severe hypoplasia of the upper part of the cerebellar vermis and the dysplasia of the cerebellar folia is evident on the coronal slices (red arrow).

He first complained of visual impairment at 25 years of age with night-blindness and reduced visual acuity. He underwent a full ophthalmological examination that included Best-Corrected Visual Acuity (BCVA) measurements, slit lamp anterior segment examination, color vision testing, fundus examination, Goldmann Visual Field (GVF) test, Optical Coherence Tomography (OCT), Fundus Autofluorescence imaging (FAF), and full-field electroretinography (ERG). GVF was measured by moving the III4e and V4e stimulus target on a calibrated standard Goldmann perimeter [18]. OCT and FAF were performed with a Heidelberg Eye Explorer Version 1.9.11.0 by an experienced operator. ERG was recorded according to the International Guidelines of the International Society of Clinical Electrophysiology of Vision (ISCEV) [19]. He had a BCVA of 20/80 in the right eye (RE) (sphere −6 = cylinder −1 alpha 10°) and 20/40 in the left eye (LE) (sphere −5 = cylinder −2 alpha 180°). The Ishihara color vision test ascertained the subject’s inability to read any pseudoisochromatic plate except for the test plate. Intraocular pressure was 16 mmHg in both eyes. Under biomicroscopy, lenses were clear in both eyes. Fundus examination revealed a waxy pallor of the optic disc with a circumpapillary atrophy, and widespread dystrophy of the retinal pigment epithelium (RPE) with bone spicule-shaped pigment deposits in mid-periphery (Figure 1a,b). OCT revealed a reduced macular thickness and RPE dystrophy in both eyes, and vitreoretinal interface syndrome in the LE (Figure 1c,d). Focal areas of hypoautofluorescence at the posterior pole, with foveal hyperautofluorescence in the RE and foveal hypoautofluorescence in the LE, were detected by FAF imaging (Figure 1e,f). The GVF test showed a concentric constriction to central 10°–20° measured with a III4e and V4e target, respectively (Figure 1g,h). Scotopic and photopic ERG responses were below the noise level (Supplementary Figure S1). Based on the above findings, a clinical diagnosis of RP was formulated according to the criteria established by Hamel [20].

**Figure 2:**
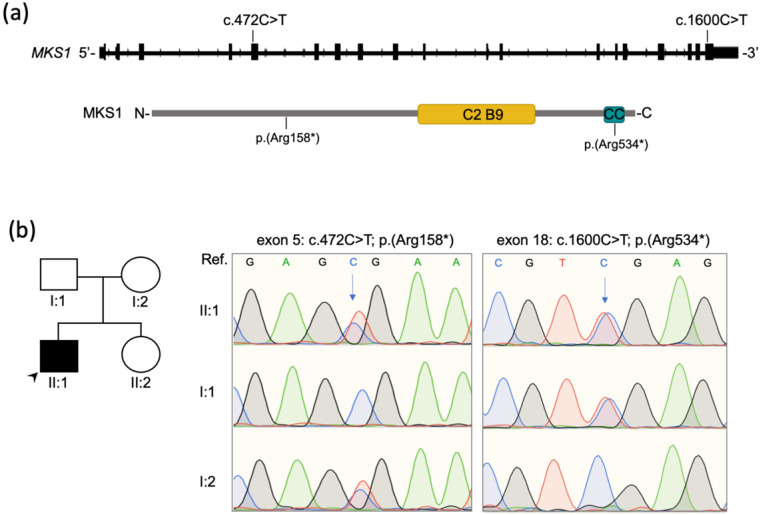
Biallelic pathogenic variants identified in *MKS1*. (**a**) Schematics showing the *MKS1* gene and protein with the relative position of the variants identified in the proband. The C2 B9-type domain (311-439 aa) and the coiled-coil domain (525-545 aa) of the MKS1 protein (UniProt Q9NXB0; 559 aa) are depicted (yellow and blue box, respectively). (**b**) Sanger sequencing traces indicate compound heterozygosity of the two variants in exon 5 and exon 18 in the patient (II:1) and heterozygosity in the unaffected parents (I:1, I:2). The arrows indicate the altered nucleotides. Arrowhead indicates the proband in the pedigree (II:1). Ref; Reference sequence.

A clinical exome was performed to investigate the defect underlying the RP. Briefly, a peripheral blood sample was collected upon written informed consent and a clinical exome library was prepared using the ClearSeq Inherited Disease Panel (Agilent; 2742 targeted genes) and run on a NextSeq500 sequencing platform (Illumina Inc., San Diego, CA, USA). The called Single Nucleotide Variants (SNV) were annotated as previously described [21]. Only variants with a minor allele frequency (MAF) < 0.05 in the Genome Aggregation Database (gnomAD; http://gnomad.broadinstitute.org; accessed in April 2021) were considered. Two nonsense variants in the *MKS1* gene (Figure 2a) were identified and validated by Sanger sequencing of the corresponding genomic fragments. Sanger sequencing in both proband’s parents showed that the variants were present *in trans* in the proband (Figure 2b).

One of the truncating variants (NM_017777: c.472C>T; p.(Arg158*)) was predicted to introduce a premature termination codon (PTC) at amino acid position 158 and was already reported in compound heterozygosity in a female patient with a complete form of MKS [8]. The other variant (NM_017777: c.1600C>T; p.(Arg534*)) was not previously reported and had an allele frequency of 7 × 10^−6^ (i.e., found in 2 out of 280,044 deposited alleles) in gnomAD only in the heterozygous state. It was not present neither in the Human Gene Mutation Database (HGMD) nor in the Leiden Open Variation Database (LOVD) but was found to have an entry in ClinVar (Variation ID: 554287). Finally, it was not reported in our internal database including over 3000 whole or clinical exomes from Italian patients. According to the American College of Medical Genetics and Genomics (ACMG) recommendations, the variant p.(Arg158*) was classified as ‘pathogenic’ based on the PVS1, PM2, PP3, PP4, PP5 criteria, whereas the variant p.(Arg534*) was categorized as ‘likely pathogenic’ based on the PM2, PM3, PP3 and PP4 criteria [22]. The variants have been deposited in LOVD v.3.0 (http://www.lovd.nl/; variant IDs: 0000784043 and 0000784042).

Because *MKS1* pathogenic variants cause syndromic ciliopathies, the patient underwent a complete physical examination to rule out extra-ocular manifestations. On physical exam, he was not noticed to have dysmorphic features or limb abnormalities such as polydactyly, and his neurologic exam was unremarkable with normal finger-to-nose-testing, muscle tone and strength. Cardiac and abdomen ultrasounds were both normal, but brain MRI performed on a 3-Tesla scanner (Siemens Trio, Erlangen, Germany) showed a malformation of the posterior cranial fossa, including hypoplastic cerebellar vermis, thickening and elongation of the superior cerebellar peduncles (‘molar tooth sign’), and cerebellar folia dysplasia (Figure 1i,j).

## 3. Discussion

The presented case is remarkable for the mild clinical presentation of Joubert syndrome in an individual carrying biallelic truncating *MSK1* variants. Although clinically recognized as an isolated RP, the identification of *MKS1* variants prompted post-genotype clinical phenotyping that revealed the typical JBTS brain malformation but no other manifestations of ciliopathies.

One of the two variants detected in the patient (p.Arg158*) is predicted to result in loss-of-function because it introduces a PTC in exon 5, whereas the other (p.Arg534*) is predicted to introduce a PTC within the last coding exon of *MKS1*. Based on the position of the premature stop codon (amino acid 534) at the terminal part of the mutated transcript, it is likely that the latter evades nonsense mediated mRNA decay [23], resulting in a putative transcript encoding a mutant protein that lacks the last 26 amino acids known to be important for proper localization and interactions of *MKS1* [24].

Biallelic *MKS1* pathogenic variants have been described in few JBTS patients who usually harbor at least one non-truncating variant [5,12,13,14,16,17]. In contrast, biallelic truncating variants in *MKS1* are associated with more severe ciliopathies, such as MKS [8,25]. To date, only two reports described biallelic *MKS1* truncating variants in individuals presenting a JBTS neurological phenotype with the distinctive ‘molar tooth sign’, hypotonia, developmental delay, intellectual disability, and dysmorphisms [16,17]. One of these patients (homozygous for the c.1461-2A>G splice-site variant, predicted *in silico* to introduce a PTC) had retinal dystrophy [17]. In the other index case, who was compound heterozygous for a frameshift (c.1058delG) and a splice-site mutation (c.191-1G>A), ophthalmological assessment was not performed [16]. Compared to these two cases, the patient herein described is significantly milder because of the lack of severe neurological manifestations, despite the presence of the pathognomonic ‘molar tooth sign’. Therefore, this case report expands the clinical spectrum of the *MSK1*-related disorders.

## 4. Conclusions

This case report expands the clinical spectrum of the *MSK1*-related disorders and illustrates the power and usefulness of exome sequencing for the accurate diagnosis of inherited retinal diseases and for revealing atypical syndromic presentations.

## Data Availability

The data generated or analyzed during this study are included in this published article. The exome analysis dataset is not publicly available according to the EU General Data Protection Regulation.

## Supplementary Materials

Supplementary material is available online at https://www.mdpi.com/article/10.3390/diagnostics11071218/s1.
