# Supplementary material for: Mild Clinical Presentation of Joubert Syndrome in a Male Adult Carrying Biallelic MKS1 Truncating Variants
Source: Diagnostics (Basel). 2021 Jul 6;11(7):1218. doi: 10.3390/diagnostics11071218 (PMC8303764; doi:10.3390/diagnostics11071218)
Supplement: Supplementary file 1 [file diagnostics-11-01218-s001.zip › diagnostics-1258729-supplementary.pdf]

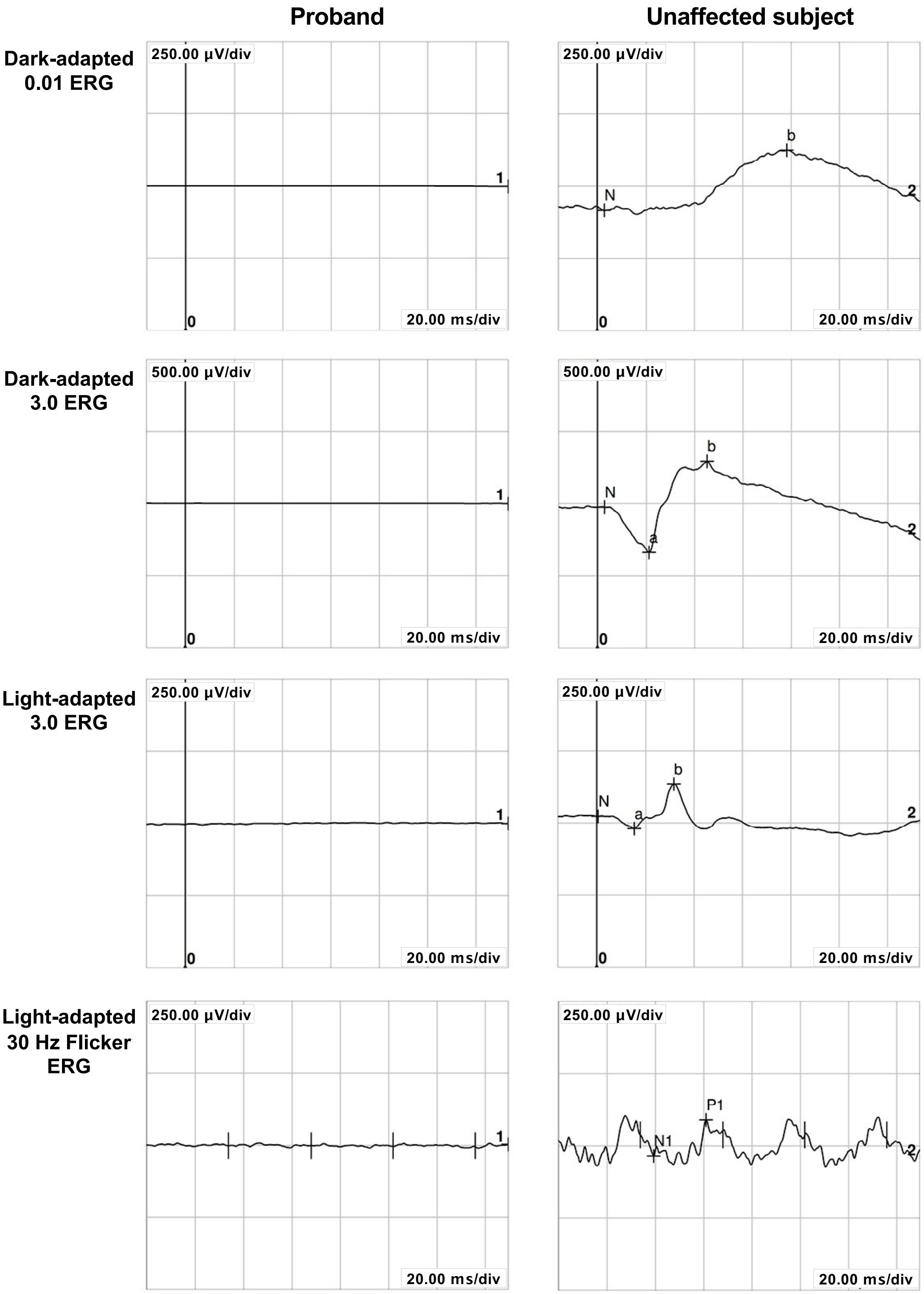

Figure S1. Full-field ERGs from the proband (panels on the left) and from a representative unaffected control subject (panels on the right). Both dark-adapted and light-adapted responses were non-recordable in the proband. Amplitude (μV/div) and latency (ms/div) scales are indicated at the upper-left and at the bottom-right corner of each image, respectively.
